# Editorial: Health Education – Fostering Public Health Literacy through Innovative Educational Activities and Resources

**DOI:** 10.3389/fpubh.2015.00208

**Published:** 2015-08-28

**Authors:** Maria João Fonseca, Graça S. Carvalho

**Affiliations:** ^1^CIBIO-InBIO, University of Porto, Porto, Portugal; ^2^CIEC, Institute of Education, University of Minho, Braga, Portugal

**Keywords:** health education, health literacy, formal and informal education, elementary school, high school

In a rapidly evolving scientifically grounded and technology-driven society, the development of health literacy is essential to ensure that the public is motivated and capable of retrieving, making sense, and applying accurate health-related information in their daily lives and professional activities. Accordingly, the investment in a sustainable public health education requires integrated actions addressed not only to lay people but also to health-specialist audiences. With the purpose of highlighting current topics in health education research, this Research Topic on “Health education: fostering public health literacy through innovative education activities and resources” brings together six articles aimed at the education of both the lay community and health professionals.

Educational interventions targeting younger population segments tend to be regarded as effective, as they increase the chance of nurturing a healthy lifestyle throughout life (1, 2). The articles by Pais et al. (3), Raved and Yarden (4), and Horn et al. (5) address key interacting challenges aimed at younger generations through school health programs. Pais et al. focus on improving the capacity of individuals and the community to deal with health-related issues (3). Raved and Yarden consider the development of the knowledge base and the system thinking skills required to process the complexity and vast amount of information about those issues (4). Horn et al. study the promotion of informed decision-making about health-related behaviors (5). Each article examines the impact of innovative methodologies and perspectives that can boost the effectiveness of health education initiatives.

Pais et al. (3) discuss the role played by the community in yielding the success of formal and informal health education initiatives. Drawing on previous studies supporting the relevance of taking on pluralistic approaches and collaborative research efforts to promote health education (6, 7), the authors present the outcomes of two mixed-methods studies carried out in a youth community organization and in a school in Portugal. By conceptualizing health according to socio-political and ecological dimensions that surpass individual and biomedical points of view and take into account the community influence, they demonstrate that young people are eager to improve their knowledge about health rights and healthcare access mainly because they perceive the great relevance of these sensitive public issues in a wider social context.

In their article, Raved and Yarden (4) present a unified model for designing teaching and learning materials and characterizing students’ systems thinking skills concerning the circulatory system, based on three well-known models: systems thinking hierarchical model, competence for cell biology education, and structure–behavior-function model (8–10). The data gathered by the implementation of this model in a sample of 75 seventh grade students (12–13 years old) in Israel suggests that this may be an efficient tool to assist educators in promoting and improving understanding about dynamic relationships established between system components at different levels of organization.

Moving beyond formal and informal educational contexts, Horn et al. (5) address the potential of large-scale evidence-based dissemination programs aimed at fostering healthy behaviors. The authors conceptualize a nine-phase dissemination model based on the Not-On-Tobacco Program, a youth smoking cessation program designed for 14–19 year olds developed in 1998 in West Virginia, USA (11), illustrating how it can be operationalized and demonstrating its applicability in other contexts.

The development of proper training actions, educational tools, and resources for health professionals must not be neglected, as they are the social actors who harness the advancements in science and technology and convert them into practices that can benefit the population’s quality of life and wellbeing (12). Two contributions are presented by Kohzaki (13, 14). These articles focus on the skills and knowledge required to fully grasp the concepts and procedures that underlie genetic testing and counseling and allow practitioners to meet the challenges presented by the rapid technological advancements in the plethora of fields that make up the health sector. Kohzaki uses the example of the typical clinical genetics education of Japanese medical professionals to outline valuable recommendations that can help promote genetic literacy and enhance the quality of the services provided.

In their opinion article, Zilberter and Zilberter (15) turn attention to healthy eating habits. Specifically, they focus on breakfast eating behavior, arguing that scarce evidence supports the assumption that this is the most important meal of the day. By reviewing relevant literature in the field, the authors conclude that the importance of this meal cannot be regarded as independent from an individual’s integrated dietary regime. The findings provide a scientific background to promote healthy eating behaviors in a wide educational context, especially in what comes to the need to critically interpret health-related information.

Taken together, these articles provide interesting insights about how to advance health education and improve health literacy to empower both lay consumers and health professionals to foster high quality decisions-making and healthy behaviors.

## Conflict of Interest Statement

The authors declare that the research was conducted in the absence of any commercial or financial relationships that could be construed as a potential conflict of interest.
